# The Relationship Between Body Fat Percentage, Anthropometric Measurements, and Diabetes Complications in Female Patients with Type 2 Diabetes

**DOI:** 10.3390/jcm14227898

**Published:** 2025-11-07

**Authors:** Ummu Nur Akinci, Cem Dogan, Egemen Tural, Akin Dayan

**Affiliations:** 1 Department of Family Medicine, Ula District Health Directorate, 48640 Mugla, Turkey; ummunur.godeoglu@gmail.com; 2 Department of Family Medicine, Derik District Health Directorate, 47800 Mardin, Turkey; 3 Department of Family Medicine, Kagithane District Health Directorate, 34408 Istanbul, Turkey; egementural1@gmail.com; 4 Department of Family Medicine, Diabetes Polyclinic, Haydarpasa Numune Training and Research Hospital, 34668 Istanbul, Turkey; akindayan@yahoo.com

**Keywords:** anthropometry, bioelectrical impedance, diabetes complications, diabetes mellitus type 2

## Abstract

**Objective**: This study aimed to investigate the relationship between body fat percentage, anthropometric measurements, and the microvascular and macrovascular complications of diabetes in female patients with type 2 diabetes mellitus. **Methods**: This was a single-center, cross-sectional study. A total of 146 female patients who met the inclusion criteria and were admitted to the diabetes clinic of a training and research hospital were enrolled after obtaining informed consent. Anthropometric measurements including height, weight, body mass index, waist circumference, hip circumference, waist-to-hip ratio, and skinfold thickness were recorded. Body fat percentage was assessed using a bioelectrical impedance analyzer. The presence of retinopathy, nephropathy, neuropathy, and macrovascular complications was documented. A *p*-value of <0.05 was considered statistically significant. **Results**: In our study, the median age of the participants was 63.0 years, and the mean duration of diabetes was 17.21 ± 8.37 years. Retinopathy was detected in 30 patients (20.5%), nephropathy in 41 patients (28.1%), neuropathy in 46 patients (31.5%), and macrovascular complications in 48 patients (32.9%). When weight was controlled as a constant variable, patients with retinopathy, nephropathy, and macrovascular complications had a statistically significant increase in waist circumference compared to those without these complications (*p* < 0.05). Suprailiac skinfold thickness was found to be positively associated with both retinopathy and nephropathy (*p* < 0.05). **Conclusions**: In our study, no significant differences were observed in body fat measurements assessed by bioimpedance analysis and skinfold thickness between female patients with and without diabetic complications. Waist circumference measurement, which can be easily performed in primary health care settings, appears to be a much more important indicator of both macrovascular and microvascular complication risk in female patients with diabetes compared to bioimpedance and skinfold thickness methods.

## 1. Background

Type 2 diabetes mellitus (T2DM) constitutes the majority of diabetes cases worldwide and is strongly linked to obesity. Central obesity, in particular, promotes insulin resistance and thereby elevates the risk of diabetic complications. Notably, approximately 90% of individuals with T2DM are overweight or obese [1,2]. Both macrovascular and microvascular complications of diabetes represent major causes of morbidity and mortality among these patients [3].

Obesity is a chronic, recurrent, and progressive disease characterized by abnormal fat accumulation that threatens health, leading to serious medical problems and an increased risk of premature death. Several methods are available to assess obesity, including anthropometric techniques such as body mass index (BMI), waist circumference (WC), hip circumference, and skinfold thickness (SFT), as well as bioelectrical impedance analysis (BIA) [2]. The practicality, low cost, and ease of application of these measurements-particularly in primary health care settings-contribute to the early identification of risk at the population level. Anthropometric assessments can be applied to large sample groups, with portable and inexpensive equipment, and the procedures are non-invasive [4]. Bioelectrical impedance analysis, on the other hand, is based on transmitting a low-level electrical current through the body, where the resistance encountered by the current is used to estimate body fat percentage [5]. BIA estimates body composition using prediction models or equations that are specific to ethnicity, sex, age, or health status. In addition to body fat percentage, BIA also provides data related to skeletal muscle mass [6]. Skeletal muscle mass is generally higher in men than in women; however, age-related muscle loss tends to follow a more linear pattern in women, whereas in men it becomes more pronounced after approximately 40 years of age [7]. Given the known differences between sexes in hormonal profile, muscle mass, and fat distribution, our study was restricted to female patients. This approach allowed us to minimize sex-related variability in the body composition measurements obtained.

Diabetic complications are major factors that reduce patients’ quality of life and increase mortality [8]. Considering the role of obesity in the development of these complications, a better understanding of the relationship between obesity and diabetic complications is crucial for clinical management [9]. Simple anthropometric measurements performed in primary health care settings enable the early identification and referral of individuals at risk for developing complications. The ease of application of anthropometric measurements in large populations, together with their cost-effectiveness and non-invasive nature, further underscores their importance for patient health.

Our literature review revealed that the number of studies simultaneously evaluating the relationship between anthropometric measurements, body fat percentage, and diabetic complications is limited [10]. The aim of this study was to investigate the association between anthropometric measurements, body fat percentage, and diabetic complications in female patients with T2DM. We further anticipate that the findings will support the prediction of complication risk using practical, low-cost methods that can be readily implemented in primary health care settings.

## 2. Materials and Methods

### 2.1. Study Design

This single-center, cross-sectional study was conducted among female patients presenting for routine follow-up at the Diabetes Clinic of a training and research hospital. The study was carried out between 1 April and 1 July 2024. All participants voluntarily agreed to take part, and written informed consent was obtained from each.

### 2.2. Inclusion and Exclusion Criteria

Female patients over 18 years of age with a diagnosis of T2DM for more than 5 years were included. Patients were excluded if they were using medications that could influence weight gain, were cachectic, pregnant, had a history of active malignancy, had physical conditions that could interfere with anthropometric measurements (e.g., limb loss, history of sudden weight loss), or had renal failure.

### 2.3. Sample Size Calculation

During the study period, 430 patients presented, of whom 200 were female. Considering these 200 women as the study population, the minimum required sample size was calculated as 132 at a 95% confidence level with a 5% margin of error. Using simple random sampling, the study was conducted with 146 participants.

### 2.4. Measures and Tools

The dependent variables were retinopathy, nephropathy, neuropathy (diabetic peripheral neuropathy), and macrovascular complications (coronary artery disease, cerebrovascular events). Anthropometric measurements, body fat percentage, demographic characteristics, and biochemical tests were evaluated as independent variables. After obtaining informed consent, demographic information—including age, education level, marital status, smoking status, chronic diseases, and regularly used medications—was recorded.

Anthropometric assessments included height, weight, WC, hip circumference, waist-to-hip ratio (WHR), BMI, SFT and body fat percentage. Weight was measured with the participant wearing light clothing and no shoes. During measurement, the participant stood upright without movement or external support, distributing weight equally on both feet. Values were recorded in kilograms (kg). Height was measured with the participant barefoot, the head positioned in the Frankfurt plane, heels together at an approximately 45° angle, and the back straight. A non-flexible, calibrated aluminum stadiometer (Seca, Hamburg, Germany) was used, and values were recorded in centimeters (cm).

BMI was calculated as weight divided by height squared (kg/m^2^). WC was measured at the midpoint between the lowest rib and the iliac crest, with the participant standing upright and without clothing over the waist. A non-elastic tape was applied without compression, and values were recorded in centimeters (cm). Hip circumference was measured with the participant standing, at the widest portion of the buttocks, keeping the tape horizontal to the floor. The WHR was computed as WC divided by hip circumference.

SFT was measured using a Cosmed Fitmate^TM^ (COSMED Srl, Rome, Italy) caliper at three sites: left triceps, suprailiac, and periumbilical (abdominal). The skinfold was grasped between the thumb and the index and middle fingers. Measurements were taken at a right angle and approximately 1 cm below the pinched skinfold and recorded in millimeters (mm). To minimize measurement error, two or three readings were obtained, and their mean value was used for analysis [5].

Body fat percentage was measured using a bioelectrical impedance analyzer (Tanita™, Tanita Corporation, Tokyo, Japan). BIA was performed in the early morning after at least 12 h of overnight fasting, in a temperature-controlled room, using a calibrated device (Tanita™, Tanita Corporation, Tokyo, Japan). Participants were barefoot, wore light clothing, and removed all metal accessories. During measurement, they stood upright with both feet placed on the footpads and both hands holding the electrodes. To ensure standardization and to minimize the influence of hydration status on the results, patients with a diagnosis of renal failure were excluded from the study.

The presence of retinopathy, nephropathy, neuropathy, and macrovascular complications was determined by reviewing the hospital information system, the national e-health portal (e-Nabız), and the Social Security Institution (SSI) medical report system. In this study, complications were defined using the following ICD-10 codes: retinopathy (H36.0, E10.3, E11.3), nephropathy (N08.3, E10.2, E11.2), neuropathy (G63.2, E10.4, E11.4), and macrovascular complications (I25.10, I70.2, E10.5, E11.5).

### 2.5. Ethical Approval

Approval was obtained from the Ethics Committee of the Training and Research Hospital on 28 August 2023 (decision no. 2023/151). The study was conducted in accordance with the Declaration of Helsinki.

### 2.6. Statistical Analysis

All statistical analyses were performed using IBM SPSS Statistics for Windows, version 21.0 (IBM Corp., Armonk, NY, USA; 2012 release). Demographic items—including age, marital status, smoking status, and chronic comorbidities—and their responses were summarized as counts (*n*) and percentages (%).

The normality of continuous variables was assessed graphically and with the Shapiro–Wilk test. Descriptive statistics for continuous variables are presented as mean ± standard deviation (SD) and median (minimum–maximum), as appropriate.

An analysis of covariance (ANCOVA) was performed to compare anthropometric and BIA measurements by diabetic complication status (retinopathy, nephropathy, neuropathy, and macrovascular complications), with body weight entered as a covariate.

The Mann–Whitney U test was used to compare HbA1c and glucose levels according to the presence of retinopathy, nephropathy, neuropathy, and macrovascular complications.

## 3. Results

Among the 146 participants, the mean age was 63.07 ± 10.91 years and the mean duration of diabetes was 17.21 ± 8.37 years. A total of 91 participants (62.3%) had at least one diabetic complication. Retinopathy was present in 30 (20.5%), nephropathy in 41 (28.1%), neuropathy in 46 (31.5%), and macrovascular complications in 48 (32.9%) participants. Demographic data, anthropometric measurements, BIA-derived indices, and biochemical parameters are presented in Table 1.

The mean age of patients with retinopathy was 66.70 ± 8.78 years, and their median diabetes duration was 20.0 (5–43) years, compared to 62.13 ± 11.24 years and 15.0 (5–40) years, respectively, in those without retinopathy; both differences were statistically significant (*p* = 0.040 and *p* = 0.010). Likewise, patients with macrovascular complications had a mean age of 67.00 ± 9.23 years and a median diabetes duration of 20 (8–40) years, whereas those without had 61.14 ± 11.19 years and 15 (5–43) years, respectively, with significant differences (*p* = 0.002 and *p* < 0.001). No significant differences were observed between groups in terms of nephropathy or neuropathy (*p* > 0.05).

After controlling for body weight, the presence of retinopathy was found to have a significant effect on WC, hip circumference, and trunk fat mass. The results of anthropometric and bioelectrical impedance measurements according to retinopathy status are presented in Table 2.

After controlling for body weight, the presence of nephropathy was found to have a significant effect on WC, WHR, and suprailiac skinfold thickness. Anthropometric and bioelectrical impedance measurements according to nephropathy status are presented in Table 3.

Anthropometric and bioelectrical impedance measurements according to neuropathy status are presented in Table 4.

After controlling for body weight, the presence of macrovascular complications was found to have a significant effect on BMI and WC. No statistically significant differences were observed in basal metabolic rate, body fat percentage, body fat mass, lean body mass, total body water, trunk fat percentage, trunk fat mass, or trunk lean mass according to the presence of macrovascular complications (*p* > 0.05). Anthropometric and bioelectrical impedance measurements by macrovascular complication status are presented in Table 5.

HbA1c levels did not differ significantly according to the presence of retinopathy. The mean HbA1c was 8.34 ± 1.98% in patients with retinopathy and 7.77 ± 1.55% in those without (z = 1.529, *p* = 0.126). Fasting plasma glucose levels were 146.33 ± 52.16 mg/dL and 157.80 ± 58.00 mg/dL, respectively (z = 0.770, *p* = 0.441).

In contrast, a statistically significant difference in HbA1c was observed with respect to nephropathy status: the mean HbA1c was 8.43 ± 1.79% in patients with nephropathy and 7.68 ± 1.56% in those without (z = 2.686, *p* = 0.007). By comparison, fasting plasma glucose levels were 160.83 ± 59.88 mg/dL and 153.34 ± 55.81 mg/dL, respectively, and did not differ significantly between groups (z = 0.533, *p* = 0.594).

HbA1c levels were similar in patients with and without neuropathy (7.78 ± 1.42% vs. 7.94 ± 1.76%; z = 0.110, *p* = 0.913). Fasting plasma glucose levels were likewise comparable between these groups, at 159.22 ± 61.45 mg/dL and 153.71 ± 54.87 mg/dL, respectively (z = 0.303, *p* = 0.762).

A similar pattern was observed for macrovascular complications. Mean HbA1c levels were 7.75 ± 1.27% in patients with macrovascular complications and 7.96 ± 1.82% in those without (z = 0.246, *p* = 0.806). Fasting plasma glucose levels were 156.00 ± 53.04 mg/dL and 155.17 ± 58.92 mg/dL, respectively, with no significant difference (z = 0.500, *p* = 0.617).

Additional comparative results are presented in the Supplementary Materials (Figure S1: Comparison of anthropometric parameters between patients with and without microvascular and macrovascular complications in T2DM).

## 4. Discussion

In this study, we investigated the relationship between anthropometric measurements, body fat percentage and diabetic complications in female patients with T2DM. When controlling for body weight, among anthropometric measurements only suprailiac skinfold thickness differed significantly between patients with and without diabetic nephropathy (DN), while among bioimpedance parameters only trunk fat mass differed between patients with and without retinopathy. No significant differences were observed in fat-free mass (FFM) between patients with and without diabetic complications. In contrast, WC showed statistically significant differences across all groups with and without both microvascular and macrovascular complications. Patients with diabetic retinopathy (DR), DN, and macrovascular complications had larger WC compared to those without these conditions. These findings support the notion that WC, a simple measurement, may serve as a more important determinant of diabetic complications than bioimpedance analysis or other anthropometric measures. Particularly in primary health care settings, WC measurement appears to be a valuable, inexpensive, rapid, and non-invasive screening tool for predicting the risk of complications.

Zhou et al. reported that each increase in WC was associated with a 1.07-fold higher risk of developing DR [11]. Similarly, Ranganathan et al. found a significant association between WC and DR [12]. Central obesity may contribute to the development of retinopathy by increasing insulin resistance and promoting inflammatory processes [13]. Consistent with these findings, patients with DR in our study had significantly larger WC.

Zhang et al. reported that a higher BMI may increase the risk of DR; however, this association did not reach statistical significance [14]. Similarly, Sen et al. found no significant relationship between BMI and DR [15]. In contrast, Huang et al. suggested that elevated BMI may be a potential risk factor for DR, DN, and diabetic peripheral neuropathy (DPN) [16]. In our study, no significant association was observed between BMI and DR. This may reflect the limitation of BMI in capturing body fat distribution, whereas measures such as WC are considered more sensitive indicators of metabolic risk [4,17]. Although BMI is widely used to categorize weight status, it does not distinguish between adiposity and lean mass, nor does it describe fat distribution. Accordingly, in primary care settings, risk assessment based solely on BMI may be insufficient; simple measures such as WC should be prioritized.

In the literature, findings regarding the association between BMI and DN remain inconsistent. Hukportie et al., in a large-scale study, reported that obesity defined by BMI may be associated with an increased risk of DN in women [18]. Conversely, Oh et al. observed no association between BMI and decline in renal function [19]. Similarly, the relationship between BMI and DPN has not been clearly established. In a meta-analysis, Liu et al. found no association between BMI and DPN [20]. However, Zhou et al. reported that higher BMI was associated with an increased risk of diabetic neuropathy, independent of sex [21].

From the standpoint of macrovascular complications, Wentworth et al. demonstrated a significant association between BMI and ischemic heart disease [22], whereas Yang et al. found no significant relationship between BMI and the incidence of cardiovascular disease [23]. These inconsistencies may reflect the inherent limitations of BMI. BMI does not account for total body fat percentage, regional fat distribution, or muscle mass. It cannot distinguish fat mass from lean mass; women and men with the same BMI may have different amounts of body fat. Individuals with high muscle mass may have an elevated BMI despite normal body fat, whereas individuals who appear lean may have a normal BMI despite excess visceral adiposity [4]. In addition, ectopic adipose tissue that is not captured by BMI can accumulate in organs such as the liver, heart, pancreas, and skeletal muscle. Such deposition is directly linked to cardiometabolic complications, yet BMI may not reliably reflect this risk [24]. Taken together, the inconsistent associations between BMI and diabetic complications suggest that both the distribution and metabolic activity of adipose tissue must be considered. Accordingly, clinical practice should emphasize not only BMI but also WC, visceral adiposity, and other anthropometric indicators.

In our study, no significant association was observed between WHR and DR, DPN, or macrovascular complications. In contrast, Wong et al. identified higher WHR as an independent predictor of DR [25], and Christensen et al. reported an association between WHR and the prevalence of DPN [26]. However, Yi et al. found no relationship between WHR and DR [27]. One possible reason we could not substantiate an association between WHR and diabetic complications is that WHR provides limited information about overall body composition; among individuals with similar waist and hip circumferences, WHR may be a misleading indicator [28].

Zheng et al. confirmed in their study that increases in both waist and hip circumference are important risk factors for DR [29]. Similarly, in Melbourne, Dirani et al. reported a significant association between WC and the presence of DR but found no relationship between triceps skinfold thickness and DR [30]. These findings are consistent with the results of our study. However, in a prospective cross-sectional case–control study conducted by Verma et al. among Indian men and women, patients with DR were found to have significantly larger WC but smaller hip circumference compared with those without DR [31]. In contrast, our study identified significantly greater values for both waist and hip circumference in patients diagnosed with DR. This discrepancy may be attributable to the fact that our study included only female patients. In women, the regional distribution of adipose tissue is influenced by hormonal differences and metabolic processes, which may account for the variation between our findings and those of previous studies.

Several studies have demonstrated that HbA1c is an independent risk marker for DN. Yang and Alrawahi reported that HbA1c is independently associated with DN, while Unnikrishnan et al. described a similar association for both microalbuminuria and overt nephropathy [32,33,34]. In our study, HbA1c levels were also significantly higher in patients with DN, which is consistent with the existing literature.

In our study, suprailiac SFT differed significantly with respect to DR. However, no significant associations were observed between mean SFT and DR, nor between abdominal, suprailiac, or mean SFT and DN. Visceral adipose tissue, compared with subcutaneous adipose tissue, is more metabolically active, shows higher rates of lipolysis, and is more resistant to insulin [35]. Adipose tissue-derived inflammatory cytokines contribute to insulin resistance and endothelial dysfunction, both of which are implicated in the development of diabetic angiopathy [36].

Visceral adipose tissue acts as a pro-inflammatory endocrine organ and affects the microvasculature through multiple mechanisms. In obesity, increased infiltration of immune cells—particularly M1 macrophages and Th1/Tc1 lymphocytes—promotes the release of cytokines such as TNF-α, IL-6, and MCP-1. This cytokine milieu drives endothelial activation and upregulates adhesion molecules (ICAM-1, VCAM-1), thereby disrupting microcirculatory integrity in tissues such as the retina and the glomerulus [37]. In addition, visceral adipose tissue produces components of the renin–angiotensin–aldosterone system (angiotensinogen/Ang II/aldosterone), which can increase intrarenal pressure, and it enhances oxidative stress through NADPH oxidase–dependent pathways. These processes facilitate proteinuria and microvascular injury [38].

Sasongko et al., using BIA-derived body composition measurements in an Indonesian cohort, reported an association between body fat percentage and DR, but not between FFM percentage and DR [39]. In contrast, we did not observe a significant association between total body fat and DR. One possible explanation is that our analyses were adjusted for body weight. The lack of a direct association between total body fat and DR in our data suggests that the absolute amount of adipose tissue may be less important than its distribution and metabolic activity. To clarify these mechanisms, future studies should incorporate both anthropometric measures and inflammatory markers in parallel.

In addition to estimating body fat, BIA has also been used in studies aiming to evaluate skeletal muscle mass and quality. In a study of 1376 Japanese individuals, Oshita et al. investigated the relationship between BIA-derived muscle quality indicators (phase angle) and age. The findings showed that these parameters exhibited marked age-related changes at the whole-body level in individuals aged ≥50 years, and specifically in the lower extremities beginning after the age of 30. In contrast, changes in the upper extremities were more limited, and in women a significant decline in phase angle in the upper limbs was observed only in those aged ≥ 85 years. These results suggest that the effect of aging on BIA parameters displays regional variation, and that lower-extremity muscle quality appears to be affected earlier and more prominently than other regions [40].

Muscle quality values are generally higher in men, which has been attributed to their greater lean mass and skeletal muscle mass. However, age-related loss of muscle mass progresses more gradually in women, whereas in men it becomes more pronounced after approximately 40 years of age [7]. These observations indicate that BIA parameters should be interpreted with consideration of both age and sex. By including only women in the present study, we aimed to reduce sex-related heterogeneity attributable to differences in muscle mass and hormonal profile, thereby enabling a clearer assessment of the relationship between BIA-derived parameters and diabetic complications in female patients.

Although several anthropometric indicators (e.g., WC and mean skinfold thickness) showed significant associations with diabetic complications, other variables such as triceps/abdominal SFT and waist-to-hip ratio did not reach statistical significance. This may reflect limited statistical power within certain complication subgroups, as well as inter-individual heterogeneity related to glycemic control, treatment regimens, and disease duration, in addition to measurement variability that is intrinsic to BIA- and SFT-based techniques. Moreover, the absence of statistical significance does not necessarily imply the absence of a biological association; some indices (e.g., BMI or waist-to-hip ratio) may simply be less sensitive to central adiposity or to patterns of fat distribution.

Our study differs from previous research by simultaneously examining the relationship between diabetic complications, anthropometric measurements, body fat analysis, and laboratory parameters. In addition, the inclusion of SFT measurements among the anthropometric assessments represents one of the strengths of this study. Several limitations should be acknowledged. Because of its cross-sectional design, it is not possible to determine the temporal sequence between adiposity-related measures and the development of specific microvascular or macrovascular complications; therefore, causality cannot be established, and the possibility of reverse causation cannot be excluded. The sample sizes within certain complication subgroups may limit the precision of some estimates. The inclusion of only female participants restricts the generalizability of the findings to male populations. In addition, although BIA is a practical and non-invasive method for assessing body composition, its accuracy may be influenced by factors such as hydration status, age-related changes in body water distribution, electrolyte imbalances, and the presence of central or abdominal obesity; these factors may have affected the measurement of body fat percentage in some participants.

## 5. Conclusions

In this study, we simultaneously evaluated the associations between anthropometric measurements, body fat analysis, and diabetic complications in female patients with T2DM. Our findings suggest that WC and central obesity may be particularly associated with DR, DN and macrovascular complications. The relationship between BMI and microvascular complications, however, was limited, likely due to the inability of BMI to adequately reflect fat distribution and metabolic risk. The assessment of SFT represents one of the strengths of our study, indicating that such measurements may provide additional insights into the role of body composition in determining complication risk.

These results indicate that simple anthropometric measurements—such as WC and SFT, which can be easily performed in primary health care settings—may serve as more valuable predictors of complication risk than bioimpedance analysis, which requires advanced technology. Future multicenter studies incorporating advanced imaging techniques will allow for a more accurate evaluation of the relationship between body fat distribution and metabolic risk. In conclusion, the assessment of complication risk in women with diabetes should not rely solely on BMI; more sensitive anthropometric indicators such as WC, visceral fat ratio, and subcutaneous fat measurements should also be taken into consideration.

## Figures and Tables

**Table 1:** Demographics, Anthropometric and Bioimpedance Measurements, and Laboratory Data.

Variable	Mean ± SD	Median (Min–Max)
Height (cm)	154.75 ± 6.32	155.0 (131–172)
Weight (kg)	77.34 ± 15.48	74.5 (48.1–141.5)
Age (years)	63.07 ± 10.91	63.0 (25–88)
Duration of diabetes (years)	17.21 ± 8.37	15.0 (5–43)
BMI (kg/m^2^)	32.31 ± 6.08	31.4 (19.3–49.3)
Waist circumference (cm)	105.90 ± 12.74	105.0 (74–148)
Hip circumference (cm)	114.88 ± 12.51	114.0 (92–159)
Waist-to-hip ratio	0.92 ± 0.06	0.92 (0.76–1.05)
Triceps SFT (mm)	23.93 ± 9.23	22.5 (5–57)
Abdominal SFT (mm)	35.61 ± 12.93	33.0 (10–75)
Suprailiac SFT (mm)	25.53 ± 9.93	24.0 (6–66)
Mean SFT (mm)	28.36 ± 9.23	27.0 (8.3–61.7)
Body fat percentage (%)	37.86 ± 6.06	38.2 (18.6–51.2)
Body fat mass (kg)	30.04 ± 10.36	28.6 (9.0–69.5)
Fat-free mass (kg)	47.32 ± 5.97	46.6 (36.7–72.0)
Trunk fat mass (kg)	13.09 ± 4.54	12.4 (3.3–28.7)

Abbreviations: SD: Standard Deviation, BMI: Body mass index, SFT: Skinfold thickness.

**Table 2:** ANCOVA Results of Anthropometric and Bioimpedance Measurements by Retinopathy Status.

Variable	Source of Variance	Sum of Squares	df	F	*p*
BMI (kg/m^2^)	Corrected Model	4339.234	2	302.984	<0.001
	Weight	4065.496	1	567.741	<0.001
	Retinopathy	14.104	1	1.970	0.163
Waist circumference (cm)	Corrected Model	17,572.504	2	210.354	<0.001
	Weight	15,649.701	1	374.673	<0.001
	Retinopathy	343.616	1	8.227	0.005
Hip circumference (cm)	Corrected Model	17,828.743	2	261.527	<0.001
	Weight	16,416.162	1	553.500	<0.001
	Retinopathy	139.250	1	4.085	0.045
Waist-to-hip ratio	Corrected Model	0.011	2	1.723	0.182
	Weight	0.006	1	1.799	0.182
	Retinopathy	0.003	1	0.992	0.321
Triceps SFT (mm)	Corrected Model	4390.717	2	39.476	<0.001
	Weight	4128.519	1	74.237	<0.001
	Retinopathy	10.979	1	0.197	0.657
Abdominal SFT (mm)	Corrected Model	4478.861	2	16.203	<0.001
	Weight	3945.874	1	28.550	<0.001
	Retinopathy	107.235	1	0.776	0.380
Suprailiac SFT (mm)	Corrected Model	3846.022	2	26.294	<0.001
	Weight	3521.332	1	48.148	<0.001
	Retinopathy	36.792	1	0.503	0.479
Mean SFT (mm)	Corrected Model	4225.771	2	37.174	<0.001
	Weight	3860.983	1	67.930	<0.001
	Retinopathy	43.273	1	0.761	0.384
Body fat percentage (%)	Corrected Model	3441.802	2	133.852	<0.001
	Weight	3381.826	1	263.039	<0.001
	Retinopathy	14.277	1	1.110	0.294
Body fat mass (kg)	Corrected Model	14,683.330	2	1179.027	<0.001
	Weight	14,200.782	1	2280.559	<0.001
	Retinopathy	3.109	1	0.499	0.481
Fat-free mass (kg)	Corrected Model	4292.874	2	347.030	<0.001
	Weight	4079.798	1	659.610	<0.001
	Retinopathy	3.324	1	0.537	0.465
Trunk fat percentage (%)	Corrected Model	2785.877	2	56.369	<0.001
	Weight	2783.771	1	112.653	<0.001
	Retinopathy	79.132	1	3.202	0.076
Trunk fat mass (kg)	Corrected Model	2305.411	2	240.501	<0.001
	Weight	2282.779	1	476.279	<0.001
	Retinopathy	21.891	1	4.567	0.034

Abbreviations: BMI: Body mass index, SFT: Skinfold thickness, df: Degrees of freedom.

**Table 3:** ANCOVA Results of Anthropometric and Bioimpedance Measurements by Nephropathy Status.

Variable	Source of Variance	Sum of Squares	df	F	*p*
BMI (kg/m^2^)	Corrected Model	4331.649	2	300.230	<0.001
	Weight	4050.581	1	561.499	<0.001
	Nephropathy	6.518	1	0.904	0.343
Waist circumference (cm)	Corrected Model	17,671.570	2	215.107	<0.001
	Weight	15,268.115	1	371.703	<0.001
	Nephropathy	442.682	1	10.777	0.001
Hip circumference (cm)	Corrected Model	17,691.790	2	252.426	<0.001
	Weight	16,771.008	1	478.576	<0.001
	Nephropathy	2.297	1	0.066	0.798
Waist-to-hip ratio	Corrected Model	0.037	2	6.159	0.003
	Weight	0.002	1	0.807	0.371
	Nephropathy	0.029	1	9.717	0.002
Triceps SFT (mm)	Corrected Model	4392.835	2	39.505	<0.001
	Weight	4275.546	1	76.901	<0.001
	Nephropathy	13.097	1	0.236	0.628
Abdominal SFT (mm)	Corrected Model	4588.647	2	16.693	<0.001
	Weight	3763.242	1	27.381	<0.001
	Nephropathy	217.021	1	1.579	0.211
Suprailiac SFT (mm)	Corrected Model	4181.142	2	29.541	<0.001
	Weight	3142.385	1	44.394	<0.001
	Nephropathy	372.912	1	5.268	0.023
Mean SFT (mm)	Corrected Model	4285.341	2	37.976	<0.001
	Weight	3712.458	1	65.799	<0.001
	Nephropathy	102.843	1	1.823	0.179
Body fat percentage (%)	Corrected Model	3428.928	2	132.424	<0.001
	Weight	3295.532	1	254.544	<0.001
	Nephropathy	1.403	1	0.108	0.743
Body fat mass (kg)	Corrected Model	14,686.516	2	1183.517	<0.001
	Weight	14,117.883	1	2275.387	<0.001
	Nephropathy	6.295	1	1.015	0.316
Fat-free mass (kg)	Corrected Model	4295.278	2	348.170	<0.001
	Weight	4021.368	1	651.934	<0.001
	Nephropathy	5.728	1	0.929	0.337
Trunk fat percentage (%)	Corrected Model	2711.541	2	53.735	<0.001
	Weight	2627.76	1	104.149	<0.001
	Nephropathy	4.797	1	0.190	0.663
Trunk fat mass (kg)	Corrected Model	2291.428	2	234.263	<0.001
	Weight	2233.276	1	456.635	<0.001
	Nephropathy	7.908	1	1.617	0.206

Abbreviations: BMI: Body mass index, SFT: Skinfold thickness, df: Degrees of freedom.

**Table 4:** ANCOVA Results of Anthropometric and Bioimpedance Measurements by Neuropathy Status.

Variable	Source of Variance	Sum of Squares	df	F	*p*
BMI (kg/m^2^)	Corrected Model	4328.483	2	299.093	<0.001
	Weight	4182.007	1	577.943	<0.001
	Neuropathy	3.352	1	0.463	0.497
Waist circumference (cm)	Corrected Model	17,269.657	2	196.753	<0.001
	Weight	16,548.597	1	377.075	<0.001
	Neuropathy	40.769	1	0.929	0.337
Hip circumference (cm)	Corrected Model	17,694.574	2	252.606	<0.001
	Weight	17,163.482	1	490.048	<0.001
	Neuropathy	5.081	1	0.145	0.704
Waist-to-hip ratio	Corrected Model	0.009	2	1.365	0.259
	Weight	0.007	1	2.129	0.147
	Neuropathy	0.001	1	0.288	0.592
Triceps SFT (mm)	Corrected Model	4380.911	2	39.339	<0.001
	Weight	4250.294	1	76.333	<0.001
	Neuropathy	1.173	1	0.021	0.885
Abdominal SFT (mm)	Corrected Model	4443.088	2	16.045	<0.001
	Weight	4093.438	1	29.564	<0.001
	Neuropathy	71.462	1	0.516	0.474
Suprailiac SFT (mm)	Corrected Model	3848.640	2	26.318	<0.001
	Weight	3836.664	1	52.473	<0.001
	Neuropathy	39.410	1	0.539	0.464
Mean SFT (mm)	Corrected Model	4183.678	2	36.614	<0.001
	Weight	4058.324	1	71.034	<0.001
	Neuropathy	1.180	1	0.021	0.886
Body fat percentage (%)	Corrected Model	3448.580	2	134.612	<0.001
	Weight	3427.122	1	267.548	<0.001
	Neuropathy	21.054	1	1.644	0.202
Body fat mass (kg)	Corrected Model	14,688.321	2	1186.075	<0.001
	Weight	14,427.346	1	23,330.003	<0.001
	Neuropathy	8.100	1	1.308	0.255
Fat-free mass (kg)	Corrected Model	4298.126	2	349.529	<0.001
	Weight	4125.351	1	670.958	<0.001
	Neuropathy	8.576	1	1.395	0.240
Trunk fat percentage (%)	Corrected Model	2763.943	2	55.581	<0.001
	Weight	2763.489	1	111.143	<0.001
	Neuropathy	57.198	1	2.300	0.132
Trunk fat mass (kg)	Corrected Model	2297.113	2	236.769	<0.001
	Weight	2282.378	1	470.500	<0.001
	Neuropathy	13.593	1	2.802	0.096

Abbreviations: BMI: Body mass index, SFT: Skinfold thickness, df: Degrees of freedom.

**Table 5:** ANCOVA Results of Anthropometric and Bioimpedance Measurements by Presence of Macrovascular Complications.

Variable	Source of Variance	Sum of Squares	df	F	*p*
BMI (kg/m^2^)	Corrected Model	4357.414	2	309.752	<0.001
	Weight	4339.368	1	616.939	<0.001
	Macrovascular Complications	32.283	1	4.590	0.034
Waist circumference (cm)	Corrected Model	17,590.358	2	211.199	<0.001
	Weight	17,589.561	1	422.379	<0.001
	Macrovascular Complications	361.470	1	8.680	0.004
Hip circumference (cm)	Corrected Model	17,795.712	2	259.285	<0.001
	Weight	17,700.415	1	515.794	<0.001
	Macrovascular Complications	106.219	1	3.095	0.081
Waist-to-hip ratio	Corrected Model	0.014	2	2.180	0.117
	Weight	0.010	1	3.096	0.081
	Macrovascular Complications	0.006	1	1.890	0.171
Triceps SFT (mm)	Corrected Model	4474.530	2	40.658	<0.001
	Weight	4474.439	1	81.314	<0.001
	Macrovascular Complications	94.792	1	1.723	0.191
Abdominal SFT (mm)	Corrected Model	4482.299	2	16.218	<0.001
	Weight	4482.066	1	32.435	<0.001
	Macrovascular Complications	110.673	1	0.801	0.372
Suprailiac SFT (mm)	Corrected Model	3857.299	2	26.400	<0.001
	Weight	3851.522	1	52.720	<0.001
	Macrovascular Complications	48.070	1	0.658	0.419
Mean SFT (mm)	Corrected Model	4264.641	2	37.696	<0.001
	Weight	4264.092	1	75.383	<0.001
	Macrovascular Complications	82.143	1	1.452	0.230
Body fat percentage (%)	Corrected Model	3427.770	2	132.296	<0.001
	Weight	3341.816	1	257.958	<0.001
	Macrovascular Complications	0.244	1	0.019	0.891
Body fat mass (kg)	Corrected Model	14,680.234	2	1174.693	<0.001
	Weight	14,345.788	1	2295.863	<0.001
	Macrovascular Complications	0.013	1	0.002	0.964
Fat-free mass (kg)	Corrected Model	4289.566	2	345.470	<0.001
	Weight	4195.491	1	675.787	<0.001
	Macrovascular Complications	0.016	1	0.003	0.959
Trunk fat percentage (%)	Corrected Model	2730.765	2	54.406	<0.001
	Weight	2570.741	1	102.435	<0.001
	Macrovascular Complications	24.020	1	0.957	0.330
Trunk fat mass (kg)	Corrected Model	2294.094	2	195.712	<0.001
	Weight	2186.279	1	448.736	<0.001
	Macrovascular Complications	0.981	1	0.375	0.143

Abbreviations: BMI: Body mass index, SFT: Skinfold thickness, df: Degrees of freedom.

## Data Availability

The data presented in this study are available on request from the corresponding author.

## Supplementary Materials

The following supporting information can be downloaded at: https://www.mdpi.com/article/10.3390/jcm14227898/s1, Figure S1: Comparison of anthropometric parameters between patients with and without microvascular and macrovascular complications in T2DM.

## Abbreviations

The following abbreviations are used in this manuscript:

T2DM	Type 2 diabetes mellitus
DR	Diabetic retinopathy
DN	Diabetic nephropathy
DPN	Diabetic peripheral neuropathy
WC	Waist circumference
WHR	Waist-to-hip ratio
BIA	Bioelectrical impedance analysis
BMI	Body mass index
SFT	Skinfold thickness
SSI	Social Security Institution
FFM	Fat-free mass
