# Supplementary figures and images for: The Relationship Between Body Fat Percentage, Anthropometric Measurements, and Diabetes Complications in Female Patients with Type 2 Diabetes
Source: J Clin Med. 2025 Nov 7;14(22):7898. doi: 10.3390/jcm14227898 (PMC12653822; doi:10.3390/jcm14227898)

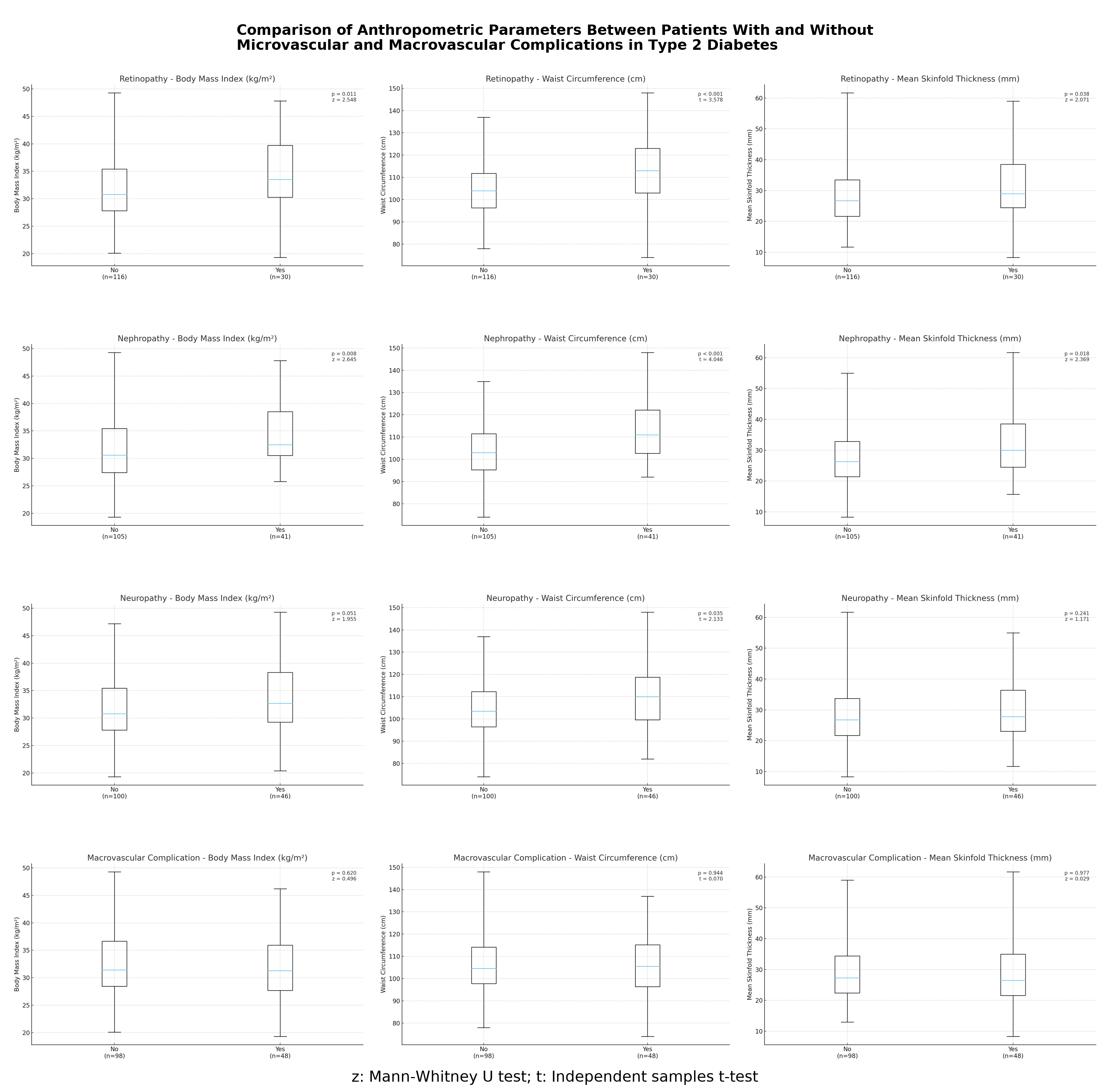

Supplement: Supplementary file 1 [file jcm-14-07898-s001.zip › jcm-3943055-supplementary.png]
